# A review of the most promising biomarkers for early diagnosis and prognosis prediction of tongue squamous cell carcinoma

**DOI:** 10.1038/s41416-018-0233-4

**Published:** 2018-08-21

**Authors:** Aisha A. Hussein, Tymour Forouzanfar, Elisabeth Bloemena, JGAM de Visscher, Ruud H. Brakenhoff, C. René Leemans, Marco N. Helder

**Affiliations:** 1 Department of Oral and Maxillofacial Surgery and Oral Pathology, Academic Centre for Dentistry, VU University Medical Center, Amsterdam, The Netherlands; 2 Department of Otolaryngology-Head and Neck Surgery, VU University Medical Center, Amsterdam, The Netherlands

**Keywords:** Prognostic markers, Oral cancer

## Abstract

**Background:**

There is a great interest in developing biomarkers to enhance early detection and clinical management of tongue squamous cell carcinoma (TSCC). However, the developmental path towards a clinically valid biomarker remains extremely challenging. Ideally, the initial key step in moving a newly discovered biomarker towards clinical implementation is independent replication. Therefore, the focus of this review is on biomarkers that consistently showed clinical relevance in two or more publications.

**Methods:**

We searched PubMed database for relevant papers across different TSCC sample sources, i.e., body fluids (saliva, serum/plasma) and tissues. No restriction regarding the date of publication was applied except for immunohistochemistry (IHC); only studies published between 2010 and June 2017 were included.

**Results:**

The search strategy identified 1429 abstracts, of which 96 papers, examining 150 biomarkers, were eventually included. Of these papers, 66% were exploratory studies evaluating single or a panel of biomarkers in one publication. Ultimately, based on studies that had undergone validation for their clinical relevance in at least two independent studies, we identified 10 promising candidates, consisting of different types of molecules (IL-6, IL-8, and Prolactin in liquid samples; HIF-1α, SOX2, E-cadherin, vimentin, MALAT1, TP53, and NOTCH1 in tissue biopsies)

**Conclusions:**

Although more exploratory research is needed with newer methods to identify biomarkers for TSCC, rigorous validation of biomarkers that have already shown unbiased assessment in at least two publications should be considered a high priority. Further research on these promising biomarkers or their combination in multi-institutional studies, could provide new possibilities to develop a specific panel for early diagnosis, prognosis, and individualized treatments.

## Background

Tongue squamous cell carcinoma TSCC is one of the most lethal head and neck cancers worldwide.^1^ It is comparatively silent and progresses from a premalignant state into invasive carcinoma without any specific alarming symptoms.^2^ This causes delay in diagnosis, eventually leading to poor prognosis. The incidence of this disease is rising in the population, particularly in Western communities among young individuals.^3–5^ Unfortunately, even with combined treatment involving surgery, radiation, and chemotherapy, the 5-year survival rate is still unsatisfactory.^6,7^ One reason could be the marked biological propensity for local invasion and the high incidence of cervical lymph node metastasis at initial diagnosis (40%).^8^ Another is a uniform treatment for all patients with the same clinical and histological features that disregards individual differences in genetic and biological behavior.

Currently, understanding of cancer development and progression is rapidly increasing. Knowledge about specific regulatory pathways and signaling interactions that lead to neoplastic transformation and invasion has been gained. Delineation of these pathways has revealed a multitude of biomolecular changes that could be exploited as biomarkers. A biomarker by definition is an objective measure such as, a gene, a protein, enzyme, or hormone that can reflect the entire spectrum of the disease, from the earliest features to the end stages. It can also provide information on how the body responds to any therapeutic interventions; this may help in making treatment decisions.^9,10^

Cancerous cells, or other body cells in response to tumor development secrete or release a subset of biomarkers into tissues and different biological body fluids. The body fluid biomarkers can be detected and evaluated in succession with non-invasive or slightly invasive means, whereas tissues-derived ones need invasive procedures like biopsies. For TSCC, finding a novel, and specific biomarker in body fluids can offer complementary information beyond what is provided by current clinical practice, especially in the field of early detection and diagnosis. Additionally, biomarkers that mirror genetic alterations and proteins expressions on histological slides may play a key role in predicting tongue cancer behavior and determining the treatment plans.

There is a three-level evidence hierarchy for biomarker validation, ranging from exploratory to validated to clinically useful, and to qualify as a useful biomarker it is essential to successfully pass them all. The exploratory biomarker is defined as any biomolecule identified in one discovery publication with targeted or untargeted approaches. This classification results in a large list of discovery biomarkers that, however, require rigorous validation. Validation is a second and pivotal step to move any biomarker towards clinical implementation, and is based primarily on confirming a discovery biomarker’s finding in at least two independent studies.^11,12^ To date, despite the proposition of a large number of potential biomarkers of TSCC, none are currently used in clinical practice, and only very few have actually proceeded towards the path of validation.

To our knowledge, this review is the first to list the published literature on both liquid and tissue-based biomarkers in TSCC. Since squamous cell carcinoma of different subsites of the oral cavity is quite heterogeneous, we only considered studies that specifically addressed the tongue locus and in particular the mobile part of the tongue. Our focus was particularly on biomarkers whose clinical significance was described in at least two independent studies. As these might represent promising biomarker candidates, we evaluated the studies with regard to the potential of these biomarkers for early diagnosis and prognosis prediction of TSCC, in which the markers demonstrated a consistent association between their expression and specific clinical outcomes. Moreover, we evaluated them using Reporting Recommendations for Tumor Marker Prognostic Studies (REMARK)^13^ guidelines for prognostic studies and STARD^14^ (Standards for Reporting of Diagnostic Accuracy) criteria for the diagnostic ones. In this way, we aim to help both researchers and clinicians in identifying and pursuing the most promising tongue cancer biomarkers for further evaluation and validation studies.

## Materials and methods

### Search strategy

Potentially eligible studies were identified in a search of US National Library of Medicine electronic database (PubMed), using combination of the following terms: “tongue carcinoma”, “tongue SCC”, “biomarker”, “ biological marker”, “tissue”, “body fluid “ “saliva”, “serum/plasma”, “ immunohistochemistry”, “long non-coding (lnc) RNA”, and “ genetic mutation”. No restriction regarding date of publication was applied except for immunohistochemistry (IHC); only studies published between 2010 and June 2017 were included to ensure that all new published evidence on potential markers since the last IHC review^15^ were encompassed. In addition, PubMed Advanced Search Builder (http://www.ncbi.nlm.nih.gov/pubmed/advanced) was utilized to identify some publications. Results were supplemented with manual searching for relevant citations. The initial search was performed in January 2017 and updated in June 2017.

One author (A.A.H.) examined all titles and abstracts to exclude studies that were beyond doubt irrelevant. Then, A.A.H. and M.N.H. assessed full-text manuscripts of all remaining studies against prespecified eligibility criteria.

### Selection of studies

#### Inclusion criteria

- Studies investigating association(s) between TSCC and biomarkers
- Studies reporting clinical significance(s) for biomarker expression
- Studies investigating biomarker expressions in oral cavity when all samples were taken from the tongue
- IHC studies encompassing multivariate analysis in statistical assessment
- English full-text version available

#### Exclusion criteria

- Studies investigating biomarkers in different anatomical subsites of oral cavity, and head and neck cancer
- Studies unclear about clinical implications
- Studies exclusively addressing the base of the tongue
- Studies investigating biomarkers only in animals
- Studies investigating micro-RNAs as biomarkers; these were already reviewed^16^ recently for their clinical implications in TSCC
- Case reports, letters to the Editor, and systematic reviews

#### Definition of the level of evidence and promising biomarkers

Biomarkers are usually classified based on the development pipeline, subdivided into 4 phases: exploratory, assay development and validation phase, retrospective validation studies, and prospective validation studies.^11,17,18^ However, since most of the TSCC biomarker studies are still in the exploratory phase with rather small sample sizes, we had to employ an alternative approach, based on the study of Teunissen and co-workers,^12^ which we slightly adapted (downscaled).

#### Ranking level of evidence (LoE)

- Negative (−): Study reported no significant association between biomarker expression and clinical values
- Weak (+): One study reported an association between biomarker expression and clinical values
- Intermediate (++): 2 independent studies reported consistent evidence of an association between biomarker expression and clinical values
- Strong (+++): ≥3 independent studies reported consistent evidence of an association between biomarker expression and clinical values

Only biomarkers with an intermediate or strong LoE, i.e., demonstrating a consistent association between their expression and specific clinical outcomes in at least two reports, were considered as promising biomarkers, even in the case that also neutral or opposite predicted outcomes were available for the same biomarker.

#### Data extraction

Included studies were classified into liquid and tissue-based biomarkers. These were further categorized according to the aforementioned LoE ranking into two groups:

- Group A: studies with negative and weak LoE
- Group B: studies with intermediate and strong LoE

Group B comprised all promising biomarkers, the master variable of interest of the current review. The studies of both groups were arranged according to year of publication, earliest to latest.

Since tissue biopsies were evaluated using various techniques, the tissue-based biomarkers were subdivided as follows:

- Protein biomarkers
- lnc RNA biomarkers
- DNA biomarkers

Information about the biomarker studied, including its usefulness, sample type and size, the method of detection, expression level, type of mutation, and validity indices were listed in table format.

### Quality assessment

For the purpose of this review, we first defined a prognostic biomarker as a marker has an association with the typical outcomes such as survival rate or recurrence or has an association with the predictor of outcomes like metastasis or tumor grade/size and differentiation. We then started screening the data and found that the vast majority of these studies were prognostic in nature, while a few were diagnostic. Consequently, the quality of the selected biomarkers studies was independently assessed by two authors (A.A.H and M.N.H) on the basis of the criteria as formulated in the Reporting Recommendations for Tumor Marker Prognostic Studies (REMARK)^13^ guidelines for prognostic studies and STARD^14^ (Standards for Reporting of Diagnostic Accuracy) criteria for the diagnostic ones. The former comprises of 20 items, and the latter consists of 30 items, in which each item can encompass several aspects in both guidelines. When all aspects of an item were clearly stated in the study, it was given 1 point, 0.5 point was attributed if some but not all aspects were mentioned, and 0 point were given when the item was not reported. Based on the total scores, the studies were subdivided into three groups: studies with a REMARK score of 15–20 or STARD score of 20–30 were assigned as high reporting quality, studies had a REMARK score of 5–14.5 or STARD score of 10–19.5 were considered to have an average reporting quality, and low reporting quality when the score ≤5 for REMARK and ≤10 for STARD. Disagreements were resolved by discussion.

## Results

**Fig. 1:**
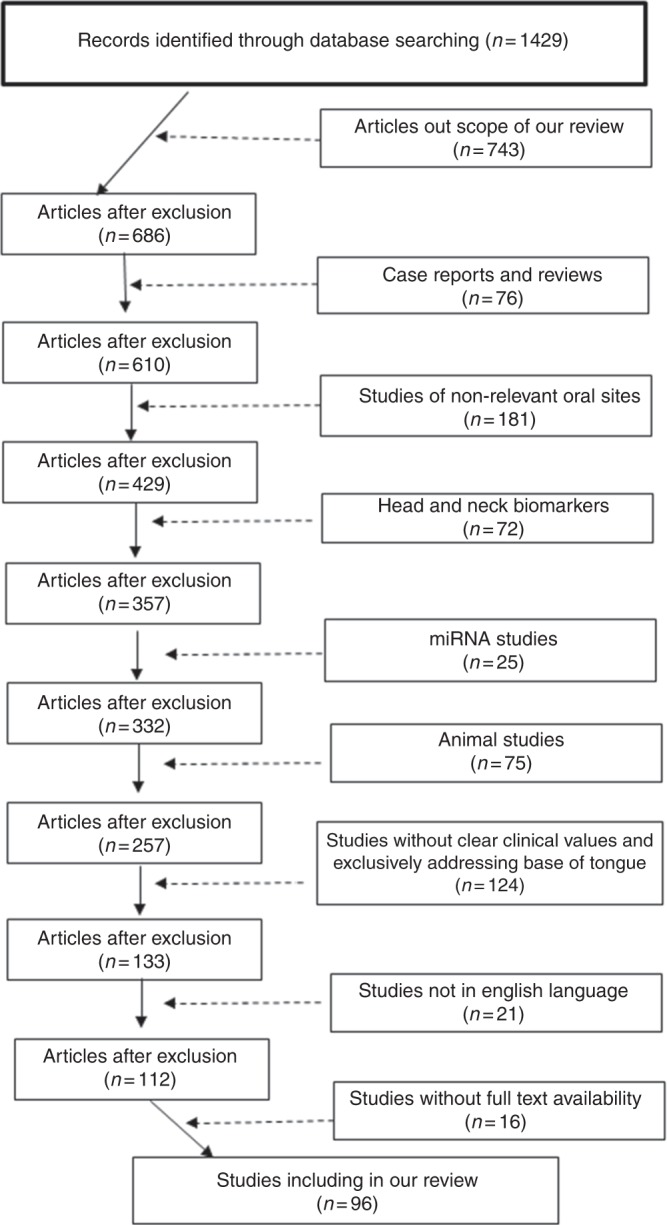
Flow chart illustrating studies selected

A diagram of studies selected for this review after exclusion of irrelevant studies is presented in Fig.(1). Seventy-two studies classified biomarkers belonging to group A, while only 24 studies satisfied the criteria for group B. In total, the included studies examined 150 biomarkers: 23 markers in body fluids, and 127 in tissue. The sample size used in these studies varied between 4 and 202 in group A, and between 17 and 248 in group B. Additionally, quality estimation according to REMARK and STARD (supplementary tables 1 and 2) showed that the overall quality of the included studies was consistent with an average rating.

In thirteen studies, the potential of salivary and blood biomarkers in tongue cancer was evaluated (Table 1). Five of these papers assessed the performance of 14 different markers for early diagnosis,^19–23^ seven assessed performance for prognosis,^24–30^ while the final study, dealing on pro-inflammatory cytokines, assessed both diagnostic and prognostic performance.^31^ Within the included studies, the most promising biomarkers were IL-6 and IL-8 that showed consistent evidence for clinical usefulness in detection and diagnosis, and prolactin in prognosis. Test accuracy indices were reported in six studies, wherein sensitivity and specificity for these studies ranged from 65%–100% and 45%–100%, respectively. In two papers^20,22^ evidence was provided that measuring a single biomarker is less effective than assessing a specific set of biomarkers, the latter showing enhanced sensitivity and specificity.

**Table 1:** Summary of body-fluid biomarkers for TSCC

Studied biomarkers	No. patients	Sample type	Significance of biomarker (s)^a^	Test accuracy indices	Expression	Potential clinical purpose	Level of evidence (LoE)^a^	References
Group A studies								
AMDL DR-70	52	Serum	AMDL DR-70	73%/93%^b^	↑	Poor prognosis	+	^24^
SCCA-1	4	Serum	SCCA-1	—	↑	Detection	+	^19^
CA125, CA19-9, TPS, CEA, SCC, and Cyfra 21-1	21	Saliva	**Cyfra 21-1, TPS, and CA125**	71%/75%^b^	↑	Detection & diagnosis	+	^20^
Adenosine deaminase	50	Saliva & serum	**Adenosine deaminase**	—	↑	Detection	+	^21^
Adiponectin	59	serum	Adiponectin	—	↓	Poor prognosis	+	^25^
** Syndecan-1**	43	Serum	**Syndecan-1**	—	↓	Progression	−	^26^
Group B studies								
Prolactin and TPS	20	Serum	**Prolactin**	100 %^c^ & 75 %	↑	Poor prognosis	+++	^27^
Prolactin, TPS, EGF, and IGF-1	52	Serum	**Prolactin**	—	↑	Poor prognosis		^28^
Prolactin	37	Serum	**Prolactin**	100%/100%^c^	↑	Poor prognosis		^29^
Prolactin	99	Serum	**Prolactin**	—	↑	Poor prognosis		^30^
IL-1a, IL-6, IL-8, VEGF-a, and TNF-a	18	Saliva	IL-1a**, IL-6, IL-8**, VEGF-a & TNF-a	—	↑	Detection & poor prognosis	++^d^	^31^
COL5A1, ABCG1, MMP1, IL-8, and FN1	37	Saliva	COL5A1, ABCG1, MMP1**, IL-8 &** FN1	65%/87%^b^	↑	Detection		^22^
IL-6	17	Serum	**IL-6**	95%/ 45%^b^	↑	Detection & diagnosis		^23^

− no significant association between biomarker and clinical value, + number of studies with statistical significant outcome = 1, ++ number of studies with consistent outcome = 2, +++ number of studies with consistent outcome ≥3, ↑increased, ↓decreased

^a^Only significant biomarker(s) used in ranking level of evidence

^b^Sensitivity/specificity

^c^Predictive value

^d^The evaluation for biomarkers in bold.

A total of 83 studies investigated different tissue-biomarkers, using various techniques (Tables 2–4). Forty-nine papers used IHC to assess expression of 82 proteins and their potential usefulness to predict prognosis (Table 2). Fifty-two proteins showed a significant association, and 13 of them were confirmed by mRNA expression. Most IHC studies belonged to group A (39, 80%). As can be deduced, five markers were independent indicators for good prognosis, while the majority (28) were adverse prognostic indicators. Group B comprised ten studies, identifying four promising IHC biomarkers: HIF-1α, SOX2, E-cadherin, and vimentin.

**Table 2:** Summary of proteins biomarkers for TSCC

Tested proteins	Sample size	Sample type	Significant biomarker^a^	Expression	Potential clinical use	Level of evidence (LoE)^a^	References
Group A studies							
Bmi-1, c-myc, and Snail	73	Tissue	**Bmi-1**	↓	Poor prognosis	+	^40^
Foxp3	81	Tissue	**Foxp3** ^b^	↑	Poor prognosis	+	^41^
RCAS1	49	Tissue	**RCAS1**	↑	Not prognosticator	−	^42^
Metallothionei	49	Tissue	**Metallothionein**	↑	Good prognosis	+	^43^
HDAC-1 and -2	49	Tissue	**HDAC-1**	↑	Not prognosticator	−	^44^
TRB3 and p-AKT	128	Tissue	**TRB3& p-AKT** ^b^	↑↓	Good prognosis,	+	^45^
MMP-2, MMP-8, MMP-9, and MMP-13	73	Tissue	**MMP-13**	↑	poor prognosis (Invasion depth & tumor size)	+	^46^
GOLPH3	179	Tissue	**GOLPH3** ^b^	↑	poor prognosis	+	^47^
FAK and Src	48	Tissue	**FAK and Src**	↑	Not Prognosticator	−	^48^
TLR5	119	Tissue	**TLR5**	↑	Poor prognosis	+	^49^
AEG-1	93	Tissue	**AEG-1** ^b^	↑	Poor prognosis	+	^50^
EZH2 and Ki-67	84	Tissue	**EZH2** ^b^	↑	poor prognosis	+	^51^
BATF2	202	Tissue	^b^ **BATF2**	↓	poor prognosis	+	^52^
FLOT1	181	Tissue	^b^ **FLOT1**	↑	Poor prognosis	+	^53^
Eph-A1, -A2, -A4, and -A7	37	Tissue	**Eph –A7**	↑	Good prognosis	+	^54^
LAT1, ASCT2, xCT, 4F2hc, and Ki-67	85	Tissue	**LAT1**	↑	Poor prognosis	+	^55^
α –SMA, N-cadherin, vimentin, and LYVE-1	50	Tissue	**α –SMA**	↑	Poor prognosis	+	^56^
p16	167	Tissue	**p16**	↑	Poor prognosis	+	^57^
t-ERK1 and p-ERK1/2	47	Tissue	**p-ERK1/2**	↑	Poor prognosis	+	^58^
PKM2 and LDH5	63	Tissue	**PKM2 & LDH5**	↑	Poor prognosis	+	^59^
LSD1 and Ki-67	63	Tissue	**LSD1** ^**b**^	↑	Poor prognosis	+	^60^
ZEB1 and CA9	84	Tissue	**ZEB1 and CA9** ^**b**^	↑	Poor prognosis	+	^61^
CAFs and Activin A	110	Tissue	**Activin A**	↑	Poor prognosis	+	^62^
MMP2 and MMP9	59	Tissue	**MMP9**	↑	Poor prognosis	+	^63^
CAF	178	Tissue	**CAF**	↑	Poor prognosis	+	^64^
Foxc2	61	Tissue	**Foxc2** ^b^	↑	Poor prognosis	+	^65^
RKIP	85	Tissue	**PKIP**	↓	poor prognosis	+	^66^
MMP13 and TLR9	195	Tissue	**TLR9**	↑	Poor prognosis	+	^67^
VEGF-C and VEGF-A	90	Tissue	**VEGF-C**	↑	Poor prognosis	+	^68^
VEGF-C, VEGFR-3, and podoplanin	65	Tissue	**VEGF-C/VEGFR-3**	↑	Not prognosticator	−	^69^
CB1R and CB2R	28	Tissue	**CB1R**	↑	Good prognosis	+	^70^
VEGF‑C, VEGFR‑3, CCR7, Nrp1,2, MVD, LVD, and SEMA3E	80	Tissue	**Nrp1**	↑	Poor prognosis	+	^71^
Securin	93	Tissue	**Securin**	↑	Not prognosticator	−	^72^
HMGA2, Snail, E-cadherin, and Vimentin	60	Tissue	^b^ **HMGA2**	↑	Poor prognosis	+	^73^
HK2	137	Tissue	**HK2** ^b^	↑	Poor prognosis	+	^74^
SUZ12	72	Tissue	**SUZ 12**	↑	Poor prognosis	+	^75^
pEGFR	48	Tissue	**pEGFR**	↑	Good prognosis	+	^76^
HA and EGFR^c^	64	Tissue	**HA**	↑	Poor prognosis	+	^77^
Nrp2, VEGF-C, VEGFR-3, and Sema3F^d^	88	Tissue	**Nrp2**	↑	Poor prognosis	+	^78^
Group B studies							
SIP1 and E-cadherin	37	Tissue	SIP1 **& E-cadherin**	↑&↓	Poor prognosis (Delayed neck metastasis)	++	^79^
Snai1,Snai2, E-cadherin, and vimentin	53 + 76 (129)	Tissue	**E-cadherin & vimentin**	↓&↑	Poor prognosis		^80^
CXCR4, CXCR12, CA9, E-cadherin, and vimentin	47	Tissue	**Vimentin**	↑	poorer prognosis	+++	^81^
Snail, Twist, E-cadherin, and Ncadherin, and vimentin	248	Tissue	**Vimentin**	↑	Poor prognosis		^82^
HIF-1α, HIF-2α TWIST2, and SNIP1	89	Tissue	**HIF-1 α**, TWIST2 & SNIP1	↑	Poor prognosis	+++	^83^
CypA, CD147, HIF-1 α, VEGF-A, and VEGF-C	80	Tissue	**HIF-1 α**	↑	Poor prognosis		^84^
HIF-1 α, CA-9, GLUT-1, and EPOR	33	Tissue	**HIF-1 α**	↑	Poor prognosis		^85^
HIF-1α and VEGF	49	Tissue	**HIF-1 α** ^b^	↑	poor prognosis		^86^
SOX2	82	Tissue	**SOX2**	↑	Poor Prognosis	++	^87^
ALDH1, CD44, OCT4, and SOX2	6	Tissue	**SOX2**	↑	Poor prognosis		^88^

− no significant association between biomarker and clinical value, + number of studies with statistical significant outcome = 1, ++ number of studies with consistent outcome = 2, +++ number of studies with consistent outcome ≥3, ↑increased, ↓decreased

^a^Only significant biomarker(s) used in ranking level of evidence

^b^studies confirmed by mRNA

^c^Electronically published in March

^d^Electronically published in June

**Table 3:** Summary of long non-coding RNAs biomarkers for TSCC

Studied biomarkers	Sample Type	Sample size		Significant biomarkers^a^	Detection method	Expression	Clinical implication	Level of evidence (LoE)^a^	References
Group A studies									
lncRNA UCA1	Tissue	94		**lncRNA UCA1**	qRT-PCR^b^	↑	Poor prognosis (Increased risk metastasis)	+	^89^
**lnc-AL355149.1-1**,lnc-PPP2R4-5, lnc-SPRR2D-1,	Tissue	32		**lnc-AL355149.1-1**	qRT-PCR	↓	Advanced T stages	+	
**lnc-MBL2-4:3**,lnc-MAN1A2-1,lnc-FAM46A-1, lnc-STXBP5-1, and lnc-MBL2-4:1				**lnc-MBL2-4:3**		↑	Poor prognosis (Increased risk metastasis)	+	^90^
lncRNA MEG3	Tissue	76		**lncRNA MEG3**	qRT-PCR	↓	Poor Prognosis	+	^91^
lncRNA HOTTIP	Tissue	86		**lncRNA HOTTIP**	qRT-PCR	↑	Poor Prognosis	+	^92^
lncRNA NKILA	Tissue	96		**lncRNA (NKILA)**	qRT-PCR	↓	Poor prognosis (Increased risk metastasis)	+	^93^
lncRNA TUG1	Tissue	27		**lncRNA (TUG1)**	qRT-PCR	↓	Detection	+	^94^
lncRNA TUC338	Tissue	25		**lncRNA TUC338**	qRT-PCR	↑	Enhanced proliferation	+	^95^
lnc RNA 152 (**LINC00152**	Tissue	15	197	**LINC00152**	qRT-PCR & in situ hybridization	↑	Detection & prognosis	+	^96^
		18 2							
lncRNA 673 (LINC00673)	Tissue	202	217	**LINC00673**	qRT-PCR	↑	Poor prognosis (Increased risk metastasis)	+	^97^
		15							
Group B studies									
MALAT1	Tissue & cell lines (CAL27 and SCC-25)	127		**MALAT1**	qRT-PCR	↑	Poor prognosis (Increased risk metastasis)	++	^98^
MALAT1	Tongue cancer cell lines and tissue	30		**MALAT1**	qRT-PCR	↑	Poor prognosis (Increased risk metastasis)		^99^

− no significant association between biomarker and clinical value, + number of studies with statistical significant outcome = 1, ++ number of studies with consistent outcome = 2, +++ number of studies with consistent outcome ≥3, ↑increased, ↓decreased

^a^Only significant biomarker(s) used in ranking level of evidence

^b^qRT-PCR: quantitative real-time polymerase chain reaction

**Table 4:** Summary of DNA biomarkers for TSCC

Studied biomarkers	Sample size	Method	Significant biomarkers^a^	Type of mutation	Potential clinical use	Prevalence	Level of evidence (LoE)^a^	References
Group A studies								
TP53	31	FISH	**TP53**	CNV (deletion)	Field cancerization	late-stage tumors 75%	+	^100^
CCND1	23	FISH	**CCND1**	(amplification) CNV	Poor prognosis	13 (56.5%)	+	^101^
CCND1	22	FISH	**CCND1**	(AmplificationCNV(	Not prognosticator	2(9.1%)	−	^102^
7q21	16	CGH	**7q21**	Copy number gain	Metastatic	44%	+	^103^
MMP-1 -1607 1G/2G and IL-8 -251 A/T	69	FISH	**MMP-1 2G/2G & IL-8 A/A**	SNP in the promoter region	Progression & recurrence	38(%53.6) 8(%116)	+	^104^
Her-2 ⁄ neu	40	FISH	**Her-2 ⁄ neu**	CNV	Not prognosticator	1(2.5%)	−	^105^
EGFR	78	FISH	**EGFR**	CNV	Not prognosticator (no correlation with survival)	35 (54%)	−	^106^
FADD	30	RT-PCR	**FADD**	CNV (Amplification)	Poor differentiation	13 (44.3%)	+	^107^
Telomeres	24	Q-FISH	**Telomeres**	Shortening	Field cancerization	—	+	^108^
WIF1 and RUNX3 methylation	76	nested methylation specific PCR method	**RUNX3**	Promotor hypermethylation	Poor prognosis (lymph node involvement)	25%	+	^109^
EGFR	89	FISH	**EGFR**	CNV	Poor prognosis	32 (36%)	+	^38^
FGFR1	123	FISH	**FGFR1**	CNV (amplification)	Not prognosticator	9.3%	−	^110^
Survivin gene	91	PCR	**Allele C**	Polymorphism at -31 G/C	Advanced stage	23% in T1 and 48% with larger tumor	+	111
TP53	115	PCR-RFLP, allele specific PCR & Sanger sequencing	**Pro72 allele**	Pro72Arg polymorphism	High risk of cancer	44 (38.3%)	+	^112^
TP53, STK11, MET, PIK3CA, BRAF and NRF2	66	Sequenom multiplexed LungCarta panel	**MET**	Missense	Poor loco-regional recurrence	10.6% (11)	+	^113^
CDKN2A	131	Sanger sequencing, FISH & Methylation-sensitive high resolution melting	**CDKN2A**	CNV missense & promotor methylation	Not prognosticator	—		^114^
8q11.21, 8q12.2-3, and 8q21.3, 22q11.23, 16p11.2, and 20q11.2	10	High density SNP array	**20q11.2**	CNV (gain)	Metastasis	50%	+	^115^
ACTN4 (protein name: actinin-4)	54	FISH	**ACTN4**	CNV (amplification)	Poor prognosis	6 (12.5%)	+	^116^
Group B studies								
TP53	39	SSCP	**TP53**	Deletion	Poor prognosis (advanced stage & high grade)	21 (54%)	+++	^117^
TP53 & CDKN2A	51	PCR, and direct sequencing on 3730xl DNA Analyzer	**TP53** & **CDKN2A**	Point mutation	Poor prognosis	10 (19.6%) 4 (7.8%)		^118^
FHIT, EGFR,LOH, TP53 DNA binding domain	121	Bidirectional sequencing, MSI &LOH analysis	**TP53 DNA binding domain**	Point mutation	Poor prognosis	18%		^119^
TP53 & NOTCH1	50	Exome sequencing, SNP genotyping, CNVs & LOH	**TP53 &**	—	Poor prognosis (nodal metastasis)	38%	++	^120^
			**NOTCH1**		Poor prognosis (Poorly differentiated tumor)	4%		
NOTCH1	60	Whole-exome & targeted deep sequencing	**NOTCH1**	Point mutation	Poor prognosis (high recurrence)	5%		^121^

− no significant association between biomarker and clinical value, + number of studies with statistical significant outcome = 1, ++ number of studies with consistent outcome = 2, +++ number of studies with consistent outcome ≥3, ↑ increased, ↓ decreased, *Q-FISH* quantitative fluorescent in situ hybridization, *RT-PCR* real-time polymerase chain reaction, *CGH* comparative genomic hybridization, *sequenom multiplexed LungCarta panel* panel of assays for somatic mutation profiling, *SNP* single nucleotide polymorphism, *SSCP* single stranded conformation polymorphism, *MSI* microsatellite instability, *LOH* loss of heterozygosity, *CNV* copy number variation

^a^Only significant biomarker(s) used in ranking level of evidence

Using quantitative RT-PCR, eleven studies evaluated lncRNA expression levels in tongue cancerous tissue (Table 3). Whereas 16 lncRNAs belonged to group A, only MALAT1 belonged to group B and thus represented the solely promising lncRNA biomarker. Studies assessing DNA mutations in TSCC evaluated 22 mutations in either a single gene or both alleles, while one evaluated promotor methylation of specific genes. Eighteen of these studies satisfied group A, and five satisfied group B, identifying TP53 and NOTCH1 as promising mutation markers.

**Fig. 2:**
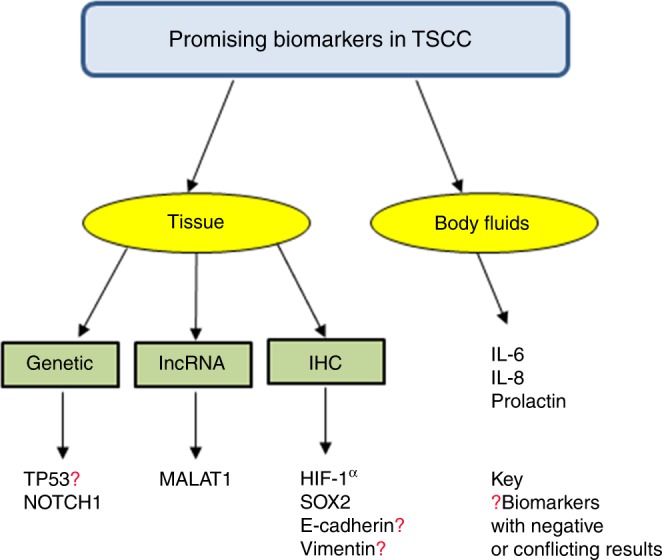
A diagram illustrating the promising biomarkers

In summary, only 22 biomarkers were evaluated in two or more independent studies, of which only 10 demonstrated a consistent association between their presence and specific clinical outcomes. Of the latter, three were biomarkers for liquid biopsies and seven were tissue-based biomarkers. Collectively, these ten biomarkers qualified as the most promising candidates for tongue cancer diagnosis and prognosis (Fig. 2).

## Discussion

Since pathology and radiology, the current keys to TSCC diagnosis and treatment decisions, are essentially visual subjective measures that are labor-intensive with limitations in diagnostic accuracy, there has been an intensified interest in biomarkers as an objective alternative and more accurate tool for early diagnosis, prognosis, or personalized treatment. A plethora of TSCC biomarker studies have been published, however, virtually all biomarkers are still in early stages of development, and far from potential application in a clinical setting. This review aimed to drive the acceleration of TSCC biomarker validation by providing an inventory of currently evaluated TSCC biomarkers across different sample sources, including saliva, serum/plasma, and tissues, and by highlighting promising biomarkers that consistently showed clinical relevance in two or more publications.

Overall, we noticed an abundance of studies that described single or multiple biomarkers only in one publication (66%), whereas there has been no corresponding increase in the validated ones. This may be due to the current pressure from journals to only publish innovative research, which prohibits researchers to perform sound repeat studies providing independent confirmation of the initial identification of a potentially promising biomarker. Since it remains in this exploratory phase pivotal to determine which biomarker is potentially promising and should be prioritized for further steps of confirmation, high-quality studies should be performed. In this regard, although we have noticed that the majority of the studied biomarkers in these discovery studies showed significant results, we observed several shortcomings affecting the reliability of their value. For example, in some publications only the data of a small number of patients are presented, while in others study designs are not the optimal or statistical design was unpowered. Two strategies should be implemented to improve this situation: one should emphasize on validation and confirmation of biomarkers that have already shown unbiased assessment in at least one publication, and the other is to conduct future research based on sound scientific and well-planned study designs so that reporting can be done according to guidelines such as REMARK for prognostic biomarkers.^13^

Last year, two other oral cancer biomarker reviews were published (Rivera et al.^32^ Almangush et al.^33^). Rivera and co-workers analyzed immunohistochemically identified potential biomarkers for oral SCC at various subsites, thereby however, disregarding the heterogeneity and well-documented variation in genomic and proteomic properties of this malignancy between different regions of the oral cavity,^34–36^ and consequently risking divergence of biomarker specificity and discriminative ability. Also, since their aim was to identify potential biomarkers per se, many biomarkers were evaluated based on one publication. Last but not least, although a scientifically sound method of biomarker evaluation was followed with a quality assessment (QA) according to REMARK guidelines, this QA only indicates the reporting quality of the study, but not necessarily the potential of the biomarker(s) at hand. Almangush et al., on the other hand, evaluated immunohistochemical biomarker studies in TSCC of three decades, and subsequently performed a meta-analysis of the five most frequently studied prognostic biomarkers. Only cyclin D1 and VEGF-A were identified as potential prognostic factors. However, they assessed the overall survival as the clinical end point based on unadjusted or “univariate” analysis, which ignored other known prognostic variables, such as tumor stage, tumor size, etc.

How does our current review relate to the two reviews described above? First of all, in contrast to both other reviews, we evaluated TSCC biomarkers across different sample sources, including saliva, serum/plasma, and tissues. Using this approach, our study identified 10 promising biomarkers, consisting of a different type of molecules: seven proteins, one lnc-RNA, and two genes (Fig. 2). Three of these markers: IL-6, IL-8, and Prolactin were detected in liquid samples, while HIF-1α, SOX2, E-cadherin, vimentin, MALAT1, TP53, and NOTCH1 were identified in tissue biopsies. Secondly, as is also the case for the Almangush review but in contrast to the Rivera report, our focus on a specific subsite within oral cancer, i.e., TSCC, is a clear advanced approach and thus our results may strongly point to unique molecular alterations. These different approaches could also explain why the Rivera paper mentioned 41 potential biomarkers, in which we merely identified ten. Thirdly, Almangush et al. did a comprehensive investigation for published prognostic biomarkers of the last 30 years, while our IHC studies were limited to the published articles in the last 7 years. Due to the technological breakthroughs in the last decade that have enabled scientists to identify new key genes and proteins in tongue carcinogenesis, we deliberately aimed to draw more attention to the latest pursued proteins such as SOX2. Last but not least, we think that a biomarker review should base its evaluation on reports employing multivariate analysis only.

Notably, these 10 promising biomarkers have demonstrated different clinical values. For example, increased expression of serum IL-6 has been found to effectively discriminate patients with TSCC from controls with an excellent sensitivity.^23^ Likewise, in another study, elevated salivary levels of IL-6 and IL-8 were reported to reliably and accurately identify the progression of TSCC from high-risk to neoplasm.^31^ This implies increased usefulness of combining these two markers in early detection of new or recurrent cases of TSCC. Nevertheless, one should be aware that increased levels of expression may be caused by sources of inflammation elsewhere, and a vigorous effort thus should be made to determine appropriate cutoff values for each marker to differentiate tongue cancer at different stages from healthy subjects. Furthermore, all biomarkers of this list showed a significant correlation with poor prognosis. In clinical practice, applicability of these biomarkers may range from recommending wider surgical resection margins to adjusting management strategy, e.g., the addition of adjuvant chemo-radiation therapy. Another key element to achieve optimal outcome may be through using them as therapeutic targets.

There is no dispute that there is an urgent and yet unmet need for novel diagnostic and prognostic biomarkers to improve TSCC treatment. Therefore, we are convinced that it is timely and highly necessary to integrate all available information about TSCC biomarkers not only from IHC samples but also from other sources. In other words: it could be important to rely on a group of molecules rather than on a single marker, because molecular evidence on multiple levels such genes, proteins, and RNAs may work in concert to prevent or promote the development of the hallmarks of cancer. Only in this sense, it will be possible to form a relatively correct picture about the molecular pathogenesis of this aggressive malignancy and identify which molecules may play a key role and accordingly, may serve as accurate biomarkers. Just as important, limiting the focus to protein expression in IHC studies only could be insufficient and misleading in the biomarker discovery phase, particularly due to the potential ongoing modifications of proteins by a plethora of post-translation changes. One such example is P53, the most frequent IHC studied protein, which has been reported to have an insignificant value in TSCC prognosis,^33^whereas we found its gene to be a strong promising indicator. Furthermore, it should be noted that as yet there is no single method suitable for reflecting the complete complexity of TSCC. Hence, our journey through different samples and various molecules assessed by different assays was in our view an essential step to find molecules with distinct biological pathways such as MALAT1 that merit further thorough investigation and validation.

Validation is a critical step for introduction of any newly discovered biomarker into the clinical practice. However, it is important to realize that there are two aspects of validation: clinical and technical. Clinical validation depends on many parameters, one of which is consistency across studies between specific clinical outcomes and the biomarker evaluated, a policy we adopted in our current study. Of equal importance are other clinical parameters that may influence the strength of a biomarker validation. These include the number of cohorts of a study, whether they are of sufficient size or not, existence of a control group, and what their characteristics are. In parallel, technical validation by using independent methods of biomarker evaluation is another parameter that should be strived for.

One major and underappreciated problem with TSCC biomarker studies which we have found is that several studies used very small samples (few with exceptions). Unfortunately, in current practice it is widely accepted that for validation studies the research must meet rigorous criteria in all aspects, particularly in sample size calculation; however, in discovery studies, such criteria are not mandatory. Indeed, neglecting this epidemiological issue in the discovery studies may have contributed to many false findings. And since the discovery studies form an essential element for the selection of biomarkers to be validated, this may partly explain why not one single biomarker has yet reached the oral oncology clinic. Admittedly, including studies with small subjects in this review may potentially bias the conclusions drawn, because the real performance of these biomarkers may remain unclear. However, we consider our validation approach for the promising biomarkers in which two or more cohorts were included as a useful strategy to minimize this bias.

One might argue that our validation approach to focus on the positive consistent studies and ignore the negative ones is considered as flawed and tenuous, particularly if these negative studies may have a higher quality. Therefore, the quality of the included studies was assessed using REMARK and STARD, which are well-established scoring systems to evaluate the quality of prognostic and diagnostic studies, respectively. Nonetheless, it should be mentioned that these two guidelines were primarily developed to assess the quality of reporting rather than to rate the research methodology. According to the evaluation in here, our results showed an average reporting quality for the included studies, which implies that these could be considered trustworthy. As such, we are confident to suggest that our list of promising biomarkers have demonstrated robustness to warrant further validation studies. Notwithstanding, we cannot speculate about the potential for clinical adoption of any of these markers. Further, we noticed that the highest scores were within lncRNA studies. Since all these studies have been published in the recent few years, this might reflect the rise in awareness among researchers about the importance of reporting and transparency in research.

The anterior two-thirds of the tongue (mobile tongue) and the posterior one-third (base of tongue) are commonly considered as two distinct clinical entities, particularly after the recognition of human papillomavirus (HPV) as a risk factor for base of the tongue in 2007.^37^ Indeed, for mobile tongue, no such link with any viruses is found in literature. To date, although each subsite of the tongue is unique with different etiological factors, pathogenesis, and prognosis, unfortunately, many authors still combine the samples of both loci or report their studies without a clear-cut specification. The scarcity of studies prohibited us to strongly apply this distinction, but we would nevertheless strongly recommend specifically addressing the tongue subsites separately.

Intriguingly, tissue-biomarkers could be investigated for its validity for detection of, and screening for TSCC in body fluids. Identification of specific biomolecules in body fluids, with a preference for saliva samples, to obtain on-the-spot potent diagnostic and prognostic information with minimal or non- invasive procedures is still a distant dream. Why this propensity for saliva? Firstly, since saliva is in direct contact with tongue cancer, accumulation of released biomarkers is likely to occur. Secondly, saliva is an ultra-filtrate of plasma, which means that blood-circulating biomolecules may be detected in saliva as well. Moreover, saliva may be preferred over serum or plasma since the latter may contain biomarker compounds derived from different sources than the actual TSCC. To evaluate the aspects listed above, biomarker levels should preferentially be simultaneously quantified in both saliva and serum/plasma samples. Finally, since biomarkers in body fluids may reflect the entire heterogeneity of cancerous tissue, a biomarker panel instead of a single biomarker may increase sensitivity and specificity.^20^ For example, a single biomarker like pro-inflammatory cytokine IL-6 or IL-8 that holds great promise is often not unique to TSCC, and no reference level of expression has been reached yet in cancer, so combining these markers, together or with other biomarkers, would likely provide a more robust clarification of true detection or prognosis.

Tissue samples are evaluated with various analytical methods, ranging from simple (such as IHC) to high technology (such as genomics) platforms. IHC is a relatively simple and affordable technique and consequently, the literature is dominated by this assay type. However, IHC suffers from considerable lack of standardization and mostly only qualitative presentation of data, making technical validation extremely difficult. Nonetheless, developments in digital pathology will improve IHC-based analyses. To solidify our results and compensate for some of these limitations, we only evaluated studies that performed multivariate analysis. Genomic approaches (e.g., microarrays, RT-PCR) are more robust and quantitative methods, with minimum analytical variability and thus facilitating technical validation. Nonetheless, these techniques cannot anticipate levels and actions of the effector molecules (proteins) in directing cancer behavior.^38^ Thus, an integral approach studying genetic mutations, RNA expression, and protein concentrations in parallel may be warranted.

Finally, it is worth mentioning that biomarker development process is financially very challenging, and moving from one phase to another becomes even more burdensome. Recently, it has been estimated that biomarker research expenditures in the U.S only in two years were over $ 2.5 billion, with nearly 500,000 publications. In contrast with this significant and massive investment in biomarker research, the number of translatable biomarkers to patients care is so far negligible.^39^ Regarding tongue cancer biomarkers, we did not find information about (industrial) financial investment, but the pattern appears similar: an overwhelming number of literature studies of potential TSCC biomarkers with no biomarker translation yet to be expected. In this view, we recommend focusing efforts on a selected set of promising biomolecules already in an early phase in order to move clinical biomarker implementation forward in an economically viable manner.

To the best of our knowledge, this is the first and largest review that evaluated specifically TSCC biomarkers across different sources, including saliva, serum/plasma, and tissues in an integral manner. The included studies used various types of assays for analysis, which allowed us to explore more details about the currently evaluated TSCC biomarkers. In addition, based on a staged approach of a biomarker validation in which one publication does not provide a meaningful role of the biomarkers as a measure of disease activity, unless more consistent evidence is available supporting its utility, we used the wide and comprehensive set of data identified here provided a shortlist of qualifying promising biomarkers. Nevertheless, our findings should be understood in the context of some limitations, which may have introduced some bias in our assessments. Firstly, we did not consider the number of patients tested in our evidence rating of the promising biomarkers due to the scarcity of the subjects in several studies. Secondly, we have included IHC studies only from 2010 onwards, consequently, it cannot be excluded that some confirmatory studies for some protein biomarkers were missed. Another limitation is that our search strategy is based on the PUBMED search engine only, which may not have revealed all relevant studies. Furthermore, validation of a biomarker such as a prolactin that emerged as one of the promising biomarkers in this review was entirely based on several studies from the same authors and this reduces the robustness of the finding. Even though, the authors followed the rule of thumb by increasing number of the patients in the confirmatory studies, further elucidation in different patient cohorts performed by different research groups to evaluate its value in forecasting prognosis should be conducted.

In conclusion, although biomarkers may play an important role in TSCC detection and management, the developmental path towards a clinically valid biomarker is always long and challenging. This study sheds some very critical light on TSCC biomarkers that demonstrated a consistent association between their expression and specific clinical outcomes at least in two publication, thus qualifying as promising candidates. Furthermore, the findings from this work show how important is the performance of the biomarker during the discovery stage because it will guide the selection of the promising markers for validation. Henceforth, it is critical at this stage to use appropriate sample size and study design. Unfortunately, two-thirds of TSCC biomarker studies have not yet advanced beyond the discovery phase. Despite the fact that more exploratory research is needed to identify specific biomarkers for TSCC, rigorous validation of biomarkers that have already shown unbiased assessment in two publications should be considered a high priority. Further research on these promising biomarkers or their combination in multi-institutional studies, could provide new possibilities to develop a specific panel that may yield better assessment of progression of this malignancy at various stages.

## Electronic supplementary material

Supplementary materials
