# Supplementary material for: A review of the most promising biomarkers for early diagnosis and prognosis prediction of tongue squamous cell carcinoma
Source: Br J Cancer. 2018 Aug 21;119(6):724–36. doi: 10.1038/s41416-018-0233-4 (PMC6173763; doi:10.1038/s41416-018-0233-4)
Supplement: Supplementary file 1 — Supplementary materials [file 41416_2018_233_MOESM1_ESM.docx]

|  | | **REMARK** | | | | | | | | | | | | | | | | | | | |  |  |
| --- | --- | --- | --- | --- | --- | --- | --- | --- | --- | --- | --- | --- | --- | --- | --- | --- | --- | --- | --- | --- | --- | --- | --- |
|  |  | **Introduction** | **Materials and Methods** | | | | | | | | | | **Results** | | | | | | | **Discussion** | |  |  |
|  |  |  | **Patiens** | | **Specimen cha** | **Assay methods** | | | | | **Statistical analysis** | | **Data** | | **Analysis and presentation** | | | | |  |  |  |  |
|  |  | **1** | **2** | **3** | **4** | **5** | **6** | **7** | **8** | **9** | **10** | **11** | **12** | **13** | **14** | **15** | **16** | **17** | **18** | **19** | **20** |  |  |
| **Biomark** | **Ref** | **State the mark** | **Describe** | **Describe** | **Describe type** | **Specify t** | **State the** | **Precisely** | **List all ca** | **Give rati** | **Specify al** | **Clarify h** | **Describe** | **Report di** | **Show the** | **Present u** | **For key** | **Among r** | **I f done, r** | **Interpret** | **Discuss** | **Score** | **Quality** |
| **AML 70** | **[24]** | **1** | **.5** | **0** | **1** | **.5** | **.5** | **1** | **1** | **0** | **.5** | **1** | **0** | **.5** | **.5** | **1** | **0** | **0** | **1** | **.5** | **0** | **10.5** | Average |
| **Adipo** | **[25]** | **1** | **.5** | **0** | **.5** | **.5** | **.5** | **1** | **1** | **0** | **.5** | **.5** | **0** | **.5** | **1** | **1** | **1** | **.5** | **0** | **1** | **1** | **12** | Average |
| **Syn** | **[26]** | **1** | **.5** | **0** | **1** | **.5** | **.5** | **1** | **1** | **0** | **.5** | **.5** | **0** | **1** | **.5** | **.5** | **0** | **0** | **0** | **1** | **1** | **10.5** | Average |
| **prol 20** | **[27]** | **1** | **.5** | **.5** | **1** | **.5** | **.5** | **1** | **1** | **0** | **.5** | **1** | **0** | **.5** | **.5** | **1** | **0** | **0** | **1** | **1** | **1** | **12.5** | Average |
| **prol52** | **[28]** | **1** | **.5** | **.5** | **1** | **.5** | **.5** | **1** | **1** | **0** | **.5** | **1** | **0** | **.5** | **.5** | **1** | **0** | **0** | **0** | **1** | **1** | **11.5** | Average |
| **Prol37** | **[29]** | **1** | **.5** | **.5** | **1** | **.5** | **.5** | **1** | **1** | **0** | **.5** | **1** | **0** | **.5** | **.5** | **1** | **0** | **0** | **0** | **1** | **1** | **11.5** | Average |
| **Prol99** | **[30]** | **1** | **.5** | **.5** | **1** | **.5** | **.5** | **1** | **1** | **0** | **.5** | **1** | **0** | **.5** | **1** | **.5** | **0** | **0** | **0** | **1** | **1** | **11.5** | Average |
| **Bmi** | **[40]** | **1** | **.5** | **.5** | **.5** | **.5** | **.5** | **1** | **1** | **0** | **.5** | **1** | **0** | **.5** | **1** | **1** | **1** | **0** | **0** | **1** | **1** | **12.5** | Average |
| **Fox3** | **[41]** | **1** | **.5** | **.5** | **.5** | **.5** | **.5** | **1** | **1** | **0** | **.5** | **1** | **0** | **.5** | **1** | **1** | **1** | **0** | **0** | **1** | **1** | **12.5** | Average |
| **RCAS** | **[42]** | **1** | **1** | **.5** | **.5** | **.5** | **.5** | **1** | **1** | **0** | **.5** | **1** | **0** | **.5** | **1** | **1** | **1** | **1** | **0** | **1** | **1** | **14** | Average |
| **Metalloth** | **[43]** | **1** | **1** | **.5** | **.5** | **.5** | **.5** | **1** | **1** | **0** | **.5** | **1** | **0** | **.5** | **1** | **1** | **1** | **1** | **0** | **1** | **1** | **14** | Average |
| **HDAC-1** | **[44]** | **1** | **1** | **.5** | **.5** | **.5** | **.5** | **1** | **1** | **0** | **.5** | **1** | **0** | **.5** | **1** | **1** | **1** | **1** | **0** | **1** | **1** | **14** | Average |
| **TRB3** | **[45]** | **1** | **.5** | **.5** | **.5** | **.5** | **.5** | **1** | **1** | **0** | **.5** | **1** | **0** | **.5** | **1** | **.5** | **0** | **0** | **0** | **1** | **1** | **11** | Average |
| **MMP-13** | **[46]** | **1** | **1** | **.5** | **.5** | **.5** | **.5** | **1** | **1** | **0** | **.5** | **1** | **.5** | **.5** | **1** | **.5** | **0** | **0** | **0** | **1** | **1** | **12** | Average |
| **GOLPH3** | **[47]** | **1** | **1** | **1** | **.5** | **.5** | **.5** | **1** | **1** | **0** | **.5** | **1** | **.5** | **.5** | **1** | **.5** | **1** | **0** | **0** | **1** | **1** | **13.5** | Average |
| **FAK and** | **[48]** | **1** | **1** | **.5** | **.5** | **.5** | **.5** | **1** | **1** | **0** | **.5** | **1** | **0** | **.5** | **1** | **1** | **0** | **0** | **0** | **1** | **1** | **12** | Average |
| **TLR5** | **[49]** | **1** | **.5** | **.5** | **.5** | **.5** | **1** | **1** | **1** | **0** | **.5** | **1** | **0** | **1** | **1** | **0** | **1** | **1** | **0** | **1** | **1** | **13.5** | Average |
| **AEG-1** | **[50]** | **1** | **.5** | **0** | **.5** | **.5** | **.5** | **1** | **1** | **0** | **.5** | **1** | **0** | **.5** | **1** | **1** | **1** | **1** | **0** | **1** | **1** | **13** | Average |
| **EZH2** | **[51]** | **1** | **1** | **.5** | **.5** | **.5** | **.5** | **1** | **1** | **0** | **.5** | **1** | **0** | **.5** | **1** | **1** | **1** | **1** | **0** | **1** | **1** | **14** | Average |
| **BATF2** | **[52]** | **1** | **.5** | **.5** | **.5** | **.5** | **.5** | **1** | **1** | **0** | **.5** | **1** | **0** | **.5** | **1** | **1** | **1** | **1** | **0** | **1** | **1** | **13.5** | Average |
| **FLOT1** | **[53]** | **1** | **1** | **.5** | **.5** | **.5** | **.5** | **1** | **1** | **0** | **.5** | **1** | **0** | **.5** | **1** | **.5** | **0** | **0** | **0** | **1** | **1** | **11.5** | Average |
| **Eph-A7** | **[54]** | **1** | **1** | **.5** | **.5** | **.5** | **.5** | **1** | **1** | **0** | **.5** | **1** | **0** | **.5** | **1** | **.5** | **0** | **0** | **0** | **1** | **1** | **11.5** | Average |
| **LAT1** | **[55]** | **1** | **.5** | **.5** | **.5** | **.5** | **.5** | **1** | **1** | **0** | **.5** | **1** | **0** | **.5** | **1** | **.5** | **1** | **1** | **0** | **1** | **1** | **13** | Average |
| **α –SMA** | **[56]** | **1** | **.5** | **.5** | **.5** | **.5** | **.5** | **1** | **1** | **0** | **.5** | **1** | **0** | **.5** | **1** | **1** | **1** | **1** | **0** | **1** | **1** | **13.5** | Average |
| **p16** | **[57]** | **1** | **1** | **1** | **.5** | **.5** | **1** | **1** | **1** | **0** | **.5** | **1** | **0** | **.5** | **1** | **1** | **1** | **1** | **0** | **1** | **1** | **15** | Average |
| **p-ERK1/** | **[58]** | **1** | **.5** | **.5** | **.5** | **.5** | **.5** | **1** | **1** | **0** | **.5** | **1** | **0** | **.5** | **1** | **1** | **1** | **1** | **0** | **1** | **1** | **13.5** | Average |
| **PKM 2 &** | **[59]** | **1** | **1** | **.5** | **.5** | **.5** | **.5** | **1** | **1** | **0** | **.5** | **1** | **0** | **.5** | **1** | **1** | **1** | **1** | **0** | **1** | **1** | **14** | Average |
| **LSD1** | **[60]** | **1** | **1** | **.5** | **.5** | **.5** | **.5** | **1** | **1** | **0** | **.5** | **1** | **0** | **.5** | **1** | **.5** | **1** | **0** | **0** | **1** | **1** | **12.5** | Average |
| **ZEB1 an** | **[61]** | **1** | **.5** | **0** | **1** | **.5** | **.5** | **1** | **1** | **0** | **.5** | **1** | **0** | **.5** | **1** | **.5** | **0** | **0** | **0** | **1** | **1** | **11** | Average |
| **Activin A** | **[62]** | **1** | **.5** | **1** | **.5** | **.5** | **.5** | **1** | **1** | **0** | **.5** | **1** | **0** | **.5** | **1** | **1** | **1** | **1** | **0** | **1** | **1** | **14** | Average |
| **MMP9** | **[63]** | **1** | **1** | **.5** | **1** | **.5** | **.5** | **1** | **1** | **0** | **.5** | **1** | **0** | **.5** | **1** | **.5** | **0** | **0** | **1** | **1** | **1** | **13** | Average |
| **CAF** | **[64]** | **1** | **.5** | **.5** | **.5** | **.5** | **.5** | **1** | **1** | **0** | **.5** | **.5** | **0** | **.5** | **1** | **.5** | **0** | **0** | **0** | **1** | **1** | **10.5** | Average |
| **Foxc2** | **[65]** | **1** | **.5** | **.5** | **.5** | **.5** | **.5** | **1** | **1** | **0** | **.5** | **1** | **0** | **.5** | **1** | **1** | **1** | **1** | **0** | **1** | **1** | **13.5** | Average |
| **PKI P** | **[66]** | **1** | **.5** | **.5** | **.5** | **.5** | **.5** | **1** | **1** | **0** | **.5** | **1** | **0** | **.5** | **1** | **1** | **1** | **1** | **0** | **1** | **1** | **13.5** | Average |
| **TLR9** | **[67]** | **1** | **.5** | **.5** | **.5** | **.5** | **.5** | **1** | **1** | **0** | **.5** | **1** | **0** | **.5** | **1** | **.5** | **1** | **0** | **0** | **1** | **1** | **12** | Average |
| **VEGF-C** | **[68]** | **1** | **.5** | **.5** | **.5** | **.5** | **.5** | **1** | **1** | **0** | **.5** | **1** | **0** | **.5** | **1** | **1** | **1** | **0** | **0** | **1** | **1** | **12.5** | Average |
| **VEGF‑C** | **[69]** | **1** | **1** | **1** | **.5** | **.5** | **.5** | **1** | **1** | **0** | **.5** | **1** | **0** | **.5** | **1** | **1** | **1** | **1** | **0** | **1** | **1** | **14.5** | Average |
| **CB1R** | **[70]** | **1** | **1** | **.5** | **.5** | **.5** | **.5** | **1** | **1** | **0** | **.5** | **1** | **0** | **.5** | **1** | **1** | **1** | **1** | **0** | **1** | **1** | **14** | Average |
| **Nrp1** | **[71]** | **1** | **.5** | **0** | **.5** | **.5** | **.5** | **1** | **1** | **0** | **.5** | **1** | **0** | **.5** | **1** | **1** | **1** | **0** | **0** | **1** | **1** | **12** | Average |
| **Securin** | **[72]** | **1** | **1** | **0** | **1** | **.5** | **.5** | **1** | **1** | **0** | **.5** | **1** | **0** | **.5** | **1** | **.5** | **1** | **0** | **0** | **1** | **1** | **12.5** | Average |
| *** HM GA2** | **[73]** | **1** | **.5** | **0** | **1** | **.5** | **.5** | **1** | **1** | **0** | **.5** | **1** | **0** | **.5** | **1** | **1** | **1** | **1** | **0** | **1** | **1** | **13.5** | Average |
| **HK2** | **[74]** | **1** | **.5** | **.5** | **.5** | **.5** | **.5** | **1** | **1** | **0** | **.5** | **1** | **0** | **.5** | **1** | **.5** | **1** | **1** | **0** | **1** | **1** | **13** | Average |
| **SUZ12** | **[75]** | **1** | **1** | **0** | **.5** | **.5** | **.5** | **1** | **1** | **0** | **.5** | **1** | **0** | **.5** | **1** | **1** | **1** | **1** | **0** | **1** | **1** | **13.5** | Average |
| **pEGFR** | **[76]** | **1** | **.5** | **.5** | **.5** | **.5** | **.5** | **1** | **1** | **0** | **.5** | **1** | **0** | **.5** | **1** | **1** | **1** | **1** | **0** | **1** | **1** | **13.5** | Average |
| **HA** | **[77]** | **1** | **1** | **1** | **.5** | **.5** | **.5** | **1** | **1** | **0** | **.5** | **1** | **0** | **.5** | **1** | **.5** | **1** | **0** | **0** | **1** | **1** | **13** | Average |
| **Nrp2** | **[78]** | **1** | **1** | **1** | **.5** | **.5** | **1** | **1** | **1** | **0** | **.5** | **1** | **0** | **.5** | **1** | **1** | **0** | **0** | **1** | **1** | **1** | **14** | Average |
| **E-cadher** | **[79]** | **1** | **1** | **.5** | **.5** | **.5** | **.5** | **1** | **1** | **0** | **.5** | **1** | **0** | **.5** | **1** | **.5** | **1** | **1** | **0** | **1** | **1** | **13.5** | Average |
| **E-cadher** | **[80]** | **1** | **.5** | **.5** | **.5** | **.5** | **.5** | **1** | **1** | **0** | **.5** | **1** | **0** | **.5** | **1** | **1** | **1** | **1** | **0** | **1** | **1** | **13.5** | Average |
| **Vimentin** | **[81]** | **1** | **1** | **.5** | **.5** | **.5** | **1** | **1** | **1** | **0** | **.5** | **1** | **0** | **.5** | **1** | **.5** | **0** | **0** | **0** | **1** | **1** | **12** | Average |
| **Vimentin** | **[82]** | **1** | **.5** | **0** | **.5** | **.5** | **.5** | **1** | **1** | **0** | **.5** | **1** | **0** | **.5** | **1** | **1** | **1** | **1** | **0** | **1** | **1** | **13** | Average |
| **HIF-1 α** | **[83]** | **1** | **1** | **0** | **.5** | **.5** | **.5** | **1** | **1** | **0** | **.5** | **1** | **0** | **.5** | **1** | **1** | **1** | **.5** | **0** | **1** | **1** | **13** | Average |
| **HIF-1 α** | **[84]** | **1** | **.5** | **0** | **.5** | **.5** | **.5** | **1** | **1** | **0** | **.5** | **1** | **0** | **.5** | **1** | **1** | **1** | **.5** | **0** | **1** | **1** | **12.5** | Average |
| **HIF-1 α** | **[85]** | **1** | **1** | **1** | **.5** | **.5** | **.5** | **1** | **1** | **0** | **.5** | **1** | **0** | **.5** | **1** | **.5** | **1** | **.5** | **0** | **1** | **1** | **13.5** | Average |
| **HIF-1 α** | **[86]** | **1** | **.5** | **1** | **.5** | **.5** | **.5** | **1** | **1** | **0** | **.5** | **1** | **0** | **.5** | **1** | **1** | **1** | **.5** | **0** | **1** | **1** | **14.5** | Average |
| **SOX2** | **[87]** | **1** | **.5** | **1** | **.5** | **.5** | **.5** | **1** | **1** | **0** | **.5** | **1** | **0** | **.5** | **1** | **1** | **1** | **1** | **0** | **1** | **1** | **14** | Average |
| **SOX2** | **[88]** | **1** | **.5** | **0** | **1** | **.5** | **.5** | **1** | **1** | **0** | **.5** | **1** | **0** | **.5** | **1** | **1** | **1** | **1** | **0** | **1** | **1** | **13.5** | Average |
| **UCA1** | **[89]** | **1** | **.5** | **.5** | **.5** | **.5** | **.5** | **1** | **1** | **0** | **.5** | **1** | **0** | **.5** | **1** | **0** | **0** | **0** | **0** | **1** | **1** | **10.5** | Average |
| **lnc-AL35** | **[90]** | **1** | **.5** | **0** | **.5** | **.5** | **.5** | **1** | **1** | **0** | **.5** | **1** | **0** | **.5** | **1** | **0** | **0** | **0** | **0** | **1** | **1** | **10** | Average |
| **MEG3** | **[91]** | **1** | **.5** | **1** | **1** | **.5** | **1** | **1** | **1** | **0** | **.5** | **1** | **1** | **.5** | **1** | **.5** | **1** | **1** | **0** | **1** | **1** | **15.5** | High |
| **HOTTIP** | **[92]** | **1** | **.5** | **1** | **1** | **.5** | **1** | **1** | **1** | **0** | **.5** | **1** | **0** | **.5** | **1** | **1** | **1** | **1** | **0** | **1** | **1** | **15** | High |
| **NKILA** | **[93]** | **1** | **.5** | **0** | **.5** | **.5** | **.5** | **1** | **1** | **0** | **.5** | **1** | **0** | **.5** | **.5** | **1** | **1** | **1** | **0** | **1** | **1** | **12.5** | Average |

| **TUC338** | **[95]** | **1** | **.5** | **.5** | **1** | **.5** | **.5** | **1** | **1** | **0** | **.5** | **1** | **0** | **.5** | **0** | **.5** | **0** | **0** | **0** | **1** | **1** | **10.5** | Average |
| --- | --- | --- | --- | --- | --- | --- | --- | --- | --- | --- | --- | --- | --- | --- | --- | --- | --- | --- | --- | --- | --- | --- | --- |
| **LINC001** | **[96]** | **1** | **.5** | **.5** | **.5** | **.5** | **.5** | **1** | **1** | **0** | **.5** | **1** | **0** | **.5** | **1** | **.5** | **0** | **0** | **0** | **1** | **1** | **11** | Average |
| **LINC006** | **[97]** | **1** | **.5** | **.5** | **.5** | **.5** | **.5** | **1** | **1** | **0** | **.5** | **1** | **0** | **.5** | **1** | **.5** | **0** | **0** | **0** | **1** | **1** | **11** | Average |
| **MALAT** | **[98]** | **1** | **.5** | **.5** | **.5** | **.5** | **.5** | **1** | **1** | **0** | **.5** | **1** | **0** | **.5** | **0** | **0** | **0** | **0** | **0** | **1** | **1** | **9.5** | Average |
| **MALAT** | **[99]** | **1** | **.5** | **.5** | **.5** | **.5** | **0** | **1** | **0** | **0** | **.5** | **1** | **0** | **.5** | **0** | **.5** | **0** | **0** | **0** | **1** | **1** | **8.5** | Average |
| **CCND1** | **[101]** | **1** | **.5** | **1** | **1** | **.5** | **.5** | **1** | **1** | **0** | **.5** | **1** | **0** | **.5** | **.5** | **0** | **0** | **0** | **0** | **1** | **1** | **11** | Average |
| **CCND1** | **[102]** | **1** | **.5** | **.5** | **.5** | **.5** | **.5** | **1** | **1** | **0** | **.5** | **1** | **0** | **.5** | **1** | **.5** | **0** | **0** | **0** | **1** | **1** | **11** | Average |
| **7q21** | **[103]** | **1** | **1** | **.5** | **.5** | **.5** | **.5** | **1** | **.5** | **0** | **.5** | **1** | **0** | **.5** | **1** | **.5** | **0** | **0** | **0** | **1** | **1** | **11** | Average |
| **MMP** | **[104]** | **1** | **1** | **1** | **.5** | **.5** | **.5** | **1** | **1** | **0** | **.5** | **1** | **0** | **.5** | **1** | **1** | **1** | **0** | **0** | **1** | **1** | **13.5** | Average |
| **Her-2** | **[105]** | **1** | **.5** | **.5** | **.5** | **.5** | **1** | **1** | **0** | **0** | **.5** | **1** | **0** | **.5** | **0** | **.5** | **0** | **0** | **0** | **1** | **1** | **9.5** | Average |
| **EGFR** | **[106]** | **1** | **.5** | **1** | **1** | **.5** | **1** | **1** | **1** | **0** | **.5** | **1** | **0** | **.5** | **1** | **.5** | **0** | **0** | **0** | **1** | **1** | **12.5** | Average |
| **FADD** | **[107]** | **1** | **.5** | **.5** | **1** | **.5** | **1** | **1** | **1** | **0** | **.5** | **1** | **0** | **.5** | **1** | **.5** | **0** | **0** | **0** | **1** | **1** | **12** | Average |
| **RUNX3** | **[109]** | **1** | **.5** | **.5** | **.5** | **1** | **.5** | **1** | **1** | **0** | **.5** | **1** | **0** | **.5** | **1** | **1** | **1** | **0** | **0** | **1** | **1** | **13** | Average |
| **EGFR** | **[38]** | **1** | **.5** | **.5** | **.5** | **.5** | **.5** | **1** | **1** | **0** | **.5** | **1** | **0** | **.5** | **1** | **.5** | **0** | **0** | **0** | **1** | **1** | **11** | Average |
| **FGFR1** | **[110]** | **1** | **.5** | **0** | **.5** | **.5** | **.5** | **1** | **1** | **0** | **.5** | **1** | **0** | **1** | **1** | **.5** | **0** | **0** | **0** | **1** | **1** | **11** | Average |
| **Allele C** | **[111]** | **1** | **.5** | **0** | **.5** | **.5** | **.5** | **.5** | **0** | **0** | **.5** | **.5** | **0** | **.5** | **1** | **.5** | **0** | **0** | **0** | **1** | **1** | **8.5** | Average |
| **M ET** | **[113]** | **1** | **1** | **.5** | **1** | **.5** | **.5** | **1** | **1** | **0** | **.5** | **1** | **0** | **1** | **1** | **.5** | **0** | **0** | **0** | **1** | **1** | **12.5** | Average |
| **CDKN2A** | **[114]** | **1** | **1** | **1** | **.5** | **.5** | **1** | **1** | **1** | **0** | **.5** | **1** | **0** | **1** | **1** | **.5** | **0** | **0** | **0** | **1** | **1** | **13** | Average |
| **20q11.2** | **[115]** | **1** | **.5** | **.5** | **.5** | **.5** | **.5** | **1** | **0** | **0** | **.5** | **0** | **0** | **.5** | **1** | **.5** | **0** | **0** | **0** | **1** | **1** | **9** | Average |
| **ACTN4** | **[116]** | **1** | **1** | **1** | **.5** | **.5** | **1** | **1** | **1** | **0** | **.5** | **.5** | **0** | **.5** | **1** | **1** | **1** | **1** | **0** | **1** | **1** | **14.5** | Average |
| **TP53** | **[117]** | **1** | **1** | **1** | **.5** | **.5** | **.5** | **1** | **1** | **0** | **0** | **.5** | **0** | **.5** | **1** | **.5** | **0** | **0** | **0** | **1** | **1** | **11** | Average |
| **TP53 & C** | **[118]** | **1** | **1** | **1** | **.5** | **.5** | **1** | **1** | **1** | **0** | **.5** | **.5** | **0** | **.5** | **1** | **1** | **1** | **1** | **0** | **1** | **1** | **14.5** | Average |
| **TP53** | **[119]** | **1** | **.5** | **0** | **1** | **.5** | **1** | **1** | **1** | **0** | **.5** | **.5** | **0** | **.5** | **1** | **.5** | **0** | **0** | **0** | **1** | **1** | **11** | Average |
| **TP53ἄ** | **[120]** |  |  |  |  |  |  |  |  |  |  |  |  |  |  |  |  |  |  |  |  |  |  |
| **NOTCH** | **[121]** | **1** | **1** | **1** | **1** | **.5** | **.5** | **1** | **1** | **0** | **.5** | **1** | **0** | **.5** | **1** | **.5** | **1** | **0** | **0** | **1** | **1** | **13.5** | Average |

ἄ: was not evaluated because we could not get the additional filesthat contain the clinical information

|  | | **STARD 2015** | | | | | | | | | | | | | | | | | | | | | | | | | | | | | | | | | |  | |
| --- | --- | --- | --- | --- | --- | --- | --- | --- | --- | --- | --- | --- | --- | --- | --- | --- | --- | --- | --- | --- | --- | --- | --- | --- | --- | --- | --- | --- | --- | --- | --- | --- | --- | --- | --- | --- | --- |
|  |  | **TITLE/ABSTRACT/ KEYWORDS** | | **Introduction** | | **Methods** | | | | | | | | | | | | | | | | | **Results** | | | | | | | | **Discussion** | | **Other info** | | |  |  |
|  |  |  | |  | | **Study**  **design** | **Participants** | | | | **Test Methods** | | | | | | | **Analysis** | | | | |  | | | | | | | |  | |  | | |  |  |
|  |  | **1** | **2** | **3** | **4** | **5** | **6** | **7** | **8** | **9** | **10a** | **10b** | **11** | **12a** | **12b** | **13a** | **13b** | **14** | **15** | **16** | **17** | **18** | **19** | **20** | **21a** | **21b** | **22** | **23** | **24** | **25** | **26** | **27** | **28** | **29** | **30** |  |  |
| **Biomarker** | **Ref** |  | | | | | | | | | | | | | | | | | | | | | | | | | | | | | | | | | | **Score** | **Quality** |
| **SCCA-1** | **[19]** | **0** | **.5** | **1** | **1** | **.5** | **0** | **1** | **0** | **0** | **1** | **0** | **0** | **0** | **0** | **0** | **0** | **0** | **.5** | **0** | **0** | **0** | **0** | **0** | **0** | **0** | **0** | **0** | **0** | **0** | **0** | **1** | **0** | **0** | **0** | **6.5** | **Low** |
| **CA125, CA19-9, TPS, CEA, SCC, & Cyfra 21- 1** | **[20]** | **1** | **1** | **1** | **1** | **.5** | **0** | **1** | **.5** | **0** | **1** | **0** | **0** | **0** | **0** | **0** | **0** | **1** | **.5** | **0** | **1** | **0** | **0** | **1** | **1** | **0** | **0** | **0** | **.5** | **0** | **0** | **1** | **0** | **0** | **0** | **13** | **Average** |
| **Adenosine deaminase** | **[21]** | **0** | **.5** | **1** | **1** | **.5** | **1** | **1** | **.5** | **0** | **1** | **0** | **0** | **0** | **0** | **0** | **0** | **0** | **.5** | **0** | **0** | **0** | **0** | **1** | **1** | **0** | **0** | **0** | **0** | **0** | **0** | **1** | **0** | **0** | **0** | **10** | **Average** |
| **IL-1a, IL-6, IL-8** | **[31]** | **0** | **.5** | **1** | **1** | **.5** | **1** | **1** | **.5** | **0** | **1** | **0** | **0** | **0** | **0** | **0** | **0** | **0** | **.5** | **0** | **0** | **0** | **0** | **1** | **1** | **0** | **0** | **0** | **0** | **0** | **0** | **1** | **0** | **0** | **0** | **10** | **Average** |
| **COL5A1, ABCG1** | **[22]** | **1** | **.5** | **1** | **1** | **.5** | **0** | **1** | **0** | **0** | **1** | **0** | **0** | **0** | **0** | **0** | **0** | **1** | **.5** | **0** | **0** | **0** | **0** | **1** | **1** | **0** | **0** | **0** | **1** | **0** | **0** | **1** | **0** | **0** | **1** | **12.5** | **average** |
| **IL-6** | **[23]** | **1** | **1** | **1** | **1** | **.5** | **1** | **1** | **.5** | **0** | **1** | **0** | **0** | **0** | **0** | **0** | **0** | **1** | **.5** | **0** | **0** | **0** | **0** | **1** | **1** | **0** | **0** | **0** | **1** | **0** | **0** | **1** | **0** | **0** | **0** | **13.5** | **Average** |
| **lncRNA TUG1** | **[94]** | **0** | **1** | **1** | **1** | **.5** | **0** | **1** | **1** | **0** | **1** | **0** | **0** | **0** | **0** | **0** | **0** | **0** | **.5** | **0** | **0** | **0** | **0** | **0** | **0** | **0** | **0** | **0** | **0** | **0** | **0** | **1** | **0** | **0** | **1** | **9** | **low** |
| **TP53** | **[100]** | **0** | **1** | **1** | **1** | **.5** | **0** | **1** | **0** | **0** | **1** | **0** | **0** | **0** | **0** | **0** | **0** | **0** | **.5** | **0** | **0** | **0** | **0** | **1** | **1** | **0** | **0** | **0** | **0** | **0** | **0** | **1** | **0** | **0** | **0** | **9** | **low** |
| **Telomer** | **[108]** | **0** | **.5** | **1** | **1** | **.5** | **0** | **1** | **0** | **0** | **1** | **0** | **0** | **0** | **0** | **0** | **0** | **0** | **.5** | **0** | **0** | **0** | **0** | **1** | **0** | **0** | **0** | **0** | **0** | **0** | **0** | **1** | **0** | **0** | **0** | **7.5** | **low** |
| **Pro72** | **[112]** | **0** | **1** | **1** | **1** | **.5** | **0** | **1** | **1** | **0** | **1** | **0** | **0** | **0** | **0** | **0** | **0** | **0** | **.5** | **0** | **0** | **0** | **0** | **1** | **0** | **0** | **0** | **0** | **0** | **0** | **0** | **1** | **0** | **1** | **1** | **11** | **average** |

| **Item:** |  |
| --- | --- |
| 1 | Identification as a study of diagnostic accuracy using at least one measure of accuracy (such as sensitivity, specificity, predictive values, or AUC) |
| 2 | Structured summary of study design, methods, results, and conclusions |
| 3 | Scientific and clinical background, including the intended use and clinical role of the index test |
| 4 | Study objectives and hypotheses |
| 5 | Whether data collection was planned before the index test and reference standard |
|  | were performed (prospective study) or after (retrospective study) |
| 6 | Eligibility criteria |
| 7 | On what basis potentially eligible participants were identified (such as symptoms, results from previous tests, inclusion in registry) |
| 8 | Where and when potentially eligible participants were identified (setting, location and dates) |
| 9 | Whether participants formed a consecutive, random or convenience series |
| 10a | Index test, in sufficient detail to allow replication |
| 10b | Reference standard, in sufficient detail to allow replication |
| 11 | Rationale for choosing the reference standard (if alternatives exist) |
| 12a | Definition of and rationale for test positivity cut-offs or result categories of the index test, distinguishing pre-specified from exploratory |
| 12b | Definition of and rationale for test positivity cut-offs or result categories of the reference standard, distinguishing pre-specified from exploratory |
| 13a | Whether clinical information and reference standard results were available to the performers/readers of the index test |
| 13b | Whether clinical information and index test results were available to the assessors of the reference standard |
| 14 | Methods for estimating or comparing measures of diagnostic accuracy |
| 15 | How indeterminate index test or reference standard results were handled |
| 16 | How missing data on the index test and reference standard were handled |
| 17 | Any analyses of variability in diagnostic accuracy, distinguishing pre-specified from exploratory |
| 18 | Intended sample size and how it was determined |
| 19 | Flow of participants, using a diagram |
| 20 | Baseline demographic and clinical characteristics of participants |
| 21a | Distribution of severity of disease in those with the target condition |
| 21b | Distribution of alternative diagnoses in those without the target condition |
| 22 | Time interval and any clinical interventions between index test and reference standard |
| 23 | Cross tabulation of the index test results (or their distribution) by the results of the reference standard |
| 24 | Estimates of diagnostic accuracy and their precision (such as 95% confidence intervals) |
| 25 | Any adverse events from performing the index test or the reference standard |
| 26 | Study limitations, including sources of potential bias, statistical uncertainty, and generalisability |
| 27 | Implications for practice, including the intended use and clinical role of the index test |
| 28 | Registration number and name of registry |
| 29 | Where the full study protocol can be accessed |
| 30 | Sources of funding and other support; role of funders |
